# Red blood cell transfusion after cardiac surgery does not result in improvement of tissue perfusion in adult patients

**DOI:** 10.1186/cc10154

**Published:** 2011-06-22

**Authors:** F Galas, JL Vincent, J Fukushima, R Nakamura, R Kalil Filho, F Jatene, JOC Auler, L Hajjar

**Affiliations:** 1 InCor, São Paulo - SP, Brazil

## Background

Most patients undergoing cardiac surgeries are exposed to red blood cell (RBC) transfusions, in the operating room or in the postoperative period. One of the main beliefs of this therapy is the ability of the RBCs to improve tissue perfusion through oxygen supply. However, recently, this concept is being questioned by some evidence as RBC storage lesion and adverse outcomes in transfused patients. The aim of this study was to determine if RBC transfusion after cardiac surgery results in improvement of tissue perfusion.

## Methods

From February 2009 to February 2010, a total of 502 patients underwent cardiac surgery with cardiopulmonary bypass at InCor - University of São Paulo. Arterial lactate, standard base deficit (SBD), arterial bicarbonate and oxygen central venous saturation (ScVO_2_) were collected immediately at the beginning and end of the procedure, immediately postoperative (POI), after 24 hours (1PO), 48 hours (2PO), 72 hours (3PO) and at ICU discharge. Mean values of these above-mentioned parameters were compared in patients exposed to RBC transfusions and patients not exposed through repeated-measures variance analysis.

## Results

**Figure 1:**
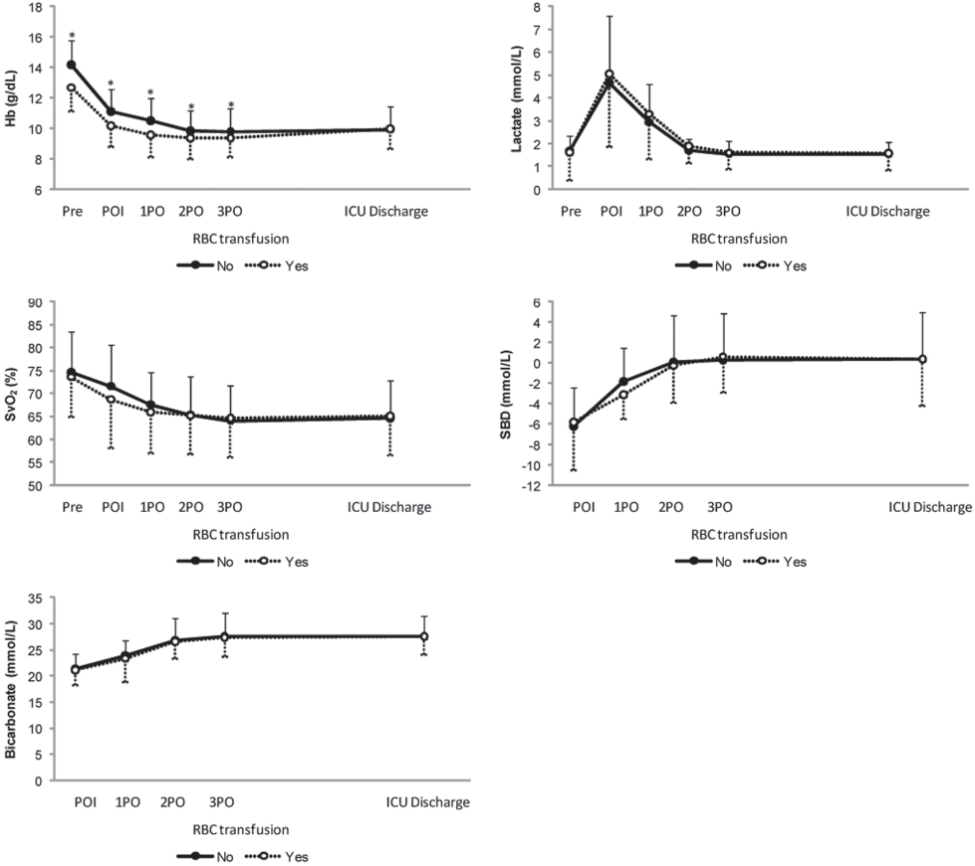
**Comparison between groups exposed or not to red blood cell transfusions considering hemoglobin (Hb) values and perfusion tissue parameters (lactate, oxygen venous central saturation, standard base excess and bicarbonate)**. *P < 0.005.

Hemoglobin values were different between groups since before surgery until just before ICU discharge and in all periods, the group not exposed to RBC transfusions presented higher values compared with the exposed group (see Figure 1 overleaf).

## Conclusion

In this prospective study, red blood transfusion did not result in improvement of tissue perfusion parameters. This finding brings to discussion the real role of blood transfusion in cardiac patients.

